# Long-term follow-up of residual masses after chemotherapy in patients with non-seminomatous germ cell tumours

**DOI:** 10.1054/bjoc.2000.1416

**Published:** 2000-10-26

**Authors:** M P Napier, A Naraghi, T J Christmas, G J S Rustin

**Affiliations:** Department of Medical Oncology, Centre for Cancer Treatment, Mount Vernon Hospital, Rickmansworth Road, Northwood, Middlesex, HA6 2RN; Department of Urology, Charing Cross Hospital, Fulham Palace Road, London, W6 8RF, UK

**Keywords:** testicular neoplasms, surgery, retroperitoneal lymph node dissection

## Abstract

This retrospective study was undertaken to determine the outcome of patients with non-seminomatous germ cell tumour who achieved a serological complete response but who had residual radiologic abnormalities upon completion of primary platinum-based chemotherapy. This was an analysis of 76 consecutive patients treated at Mount Vernon Hospital between 1983 and 1997. The patients were placed into two groups based upon whether they had surgical resection (surgery group, 48 patients) or observation (observation group, 28 patients) of residual radiologic masses on completion of initial chemotherapy (to enter the surgery group, complete surgical resection must have been achieved). The primary end-points were progression-free and overall survival. The percentage of patients alive with median follow-up 66 months was 90% for the surgery group and 80% for the observation group (*P*= 0.53, not significant). The percentage of patients continuously disease-free was 70% in the surgery group and 80% in the observation group (*P*= 0.31, not significant). In the small sub-group of patients with differentiated teratoma (TD) in the primary lesion who were observed, there was no excess risk of relapse or death. Patients who achieve a serological complete response after primary chemotherapy, but are left with ≤ 2 cm radiological masses that are not cystic and have responded, can be safely observed with diligent follow-up. © 2000 Cancer ResearchCampaign


*British Journal of Cancer* (2000) **83**(10), 1274–1280
© 2000 Cancer Research Campaign
doi: 10.1054/ bjoc.2000.1416, available online at http://www.idealibrary.com on IDEAL®

### Long-term follow-up of residual masses after chemotherapy in patients with non-seminomatous germ cell tumours

**MP Napier[1], A Naraghi[1], TJ Christmas[2] and GJS Rustin[1]**

[1]Department of Medical Oncology, Centre for Cancer Treatment, Mount Vernon Hospital, Rickmansworth Road, Northwood, Middlesex HA6 2RN; [2]Department of Urology, Charing Cross Hospital, Fulham Palace Road, London W6 8RF, UK

**Summary** This retrospective study was undertaken to determine the outcome of patients with non-seminomatous germ cell tumour who achieved a serological complete response but who had residual radiologic abnormalities upon completion of primary platinum-based chemotherapy. This was an analysis of 76 consecutive patients treated at Mount Vernon Hospital between 1983 and 1997. The patients were placed into two groups based upon whether they had surgical resection (surgery group, 48 patients) or observation (observation group, 28 patients) of residual radiologic masses on completion of initial chemotherapy (to enter the surgery group, complete surgical resection must have been achieved). The primary end-points were progression-free and overall survival. The percentage of patients alive with median follow-up 66 months was 90% for the surgery group and 80% for the observation group ($P = 0.53$, not significant). The percentage of patients continuously disease-free was 70% in the surgery group and 80% in the observation group ($P = 0.31$, not significant). In the small sub-group of patients with differentiated teratoma (TD) in the primary lesion who were observed, there was no excess risk of relapse or death. Patients who achieve a serological complete response after primary chemotherapy, but are left with ≤ 2 cm radiological masses that are not cystic and have responded, can be safely observed with diligent follow-up. © 2000 Cancer Research Campaign

**Keywords**: testicular neoplasms; surgery; retroperitoneal lymph node dissection

Platinum-based chemotherapy, complemented by surgery, has dramatically improved the prognosis of metastatic non-seminomatous germ cell tumours (NSGCT), resulting in cure rates in excess of 80% (Horwich et al, 1995). The serum markers βhCG (human chorionic gonadotropin) and αFP (alpha fetoprotein) are elevated in the majority of these cases at the time of diagnosis. It is generally accepted that those who do not achieve serological complete response (CR) after initial chemotherapy require further treatment in the form of salvage chemotherapy. However, there is no clear consensus as to the management of patients with serological CR. Treatment strategies have included surgery (usually retroperitoneal lymph node dissection (RPLND)) in all patients including those with radiological CR (Gelderman et al, 1986; Fossa et al, 1989; Aass et al, 1991), surgical intervention in those with residual masses only (Donohue and Rowland, 1984; Steyerberg et al, 1993) or resection in a selected group of patients with residual masses (Levitt et al, 1985; Hendry et al, 1993; Debono et al, 1997). Criteria proposed and used for selection of these patients have included the size of the mass, the degree of shrinkage of the mass with chemotherapy, degree of further shrinkage after chemotherapy and the histology of the primary tumour (Levitt et al, 1985, Donohue et al, 1987, Fossa et al, 1992, Hendry et al, 1993, Jaeger et al, 1994, Debono et al, 1997). This is based on work by authors examining the correlation between various factors and the histology of the residual mass. These factors have included the primary histology, marker levels, the pre- and post-chemotherapy mass size, site of the mass and the attenuation of the mass on computerized tomographic scanning (CT) and statistical models have been developed to try to predict the histology of masses post-chemotherapy (Gelderman et al, 1986; Donohue et al, 1987; Sagalowsky et al, 1990; Stomper et al, 1991; Steyerberg et al, 1994, 1995; Rabbani et al, 1996). The rationale behind this is that historically the histology of the resected material has consisted of necrosis/fibrosis in 18–49%, differentiated teratoma (TD) in 30–57% and malignancy in up to 30% (Bajorin et al, 1992; Christmas et al, 1998*a*). While resection of malignant elements or TD is beneficial, removal of necrotic or fibrotic tissue is unnecessary. Given the potential morbidity and mortality of such surgery, not to mention the financial cost, it would be advantageous to limit this intervention to those cases where it is warranted.

The best approach for radiological residual masses is not clear in patients who have achieved a serological CR. Some advocate observation if initial chemotherapy leads to >90% radiological regression (Gelderman et al, 1986). The policy at Mount Vernon Hospital has been that patients with serological and radiological CR have not been submitted to routine RPLND. The policy in patients with serological CR but residual radiological abnormalities in whom there is a high likelihood of residual TD was to perform resection, while those in whom this risk was considered low were placed on close observation with serial repeat scanning. Factors favouring observation were > 50% tumour volume reduction, absence of cystic change on CT, absence of TD in the primary tumour, and size of residual mass ≤ 2 cm. In this retrospective

*Received 10 January 2000*
*Revised 18 May 2000*
*Accepted 27 June 2000*

*Correspondence to*: GJS Rustin analysis we report our experience with 76 patients with an incomplete radiological response in the presence of normal serum tumour markers and examine the outcome in those not submitted to surgery compared to those where surgery was undertaken.

### PATIENTS AND METHODS

A retrospective analysis of the medical records of 76 patients with primary testicular or primary retroperitoneal NSGCT was performed. These patients had their cancer diagnosed between January 1980 and January 1998. The patients were identified from a database of all patients with testicular cancer referred to Mount Vernon Hospital during these times. This database was correlated with the database from the local Cancer Registrary Office (Mount Vernon site) of all patients in the district with a diagnosis of testicular cancer.

Patients were considered eligible for analysis if they had Royal Marsden Stage II, III or IV primary testicular or primary retroperitoneal NSGCT, and achieved a complete serological response (i.e. normalization of αFP and βhCG if elevated pre-chemotherapy) but who had potentially resectable residual disease on post-treatment CT scanning. On entry the following data was collected:

1. Serial pre-, on-treatment and post-chemotherapy tumour markers analysis (αFP and βhCG)
2. Results from pre-, on-treatment and post-chemotherapy CT and other radiographic examinations
3. Pathology results from diagnostic orchidectomy or biopsy, and
4. Pathology results of the resected mass in those who underwent post-chemotherapy surgery. Pathology was classified according to the terminology of the British Testicular Tumour Panel (Pugh, 1976).

No patients were excluded because of missing data.

Patients were excluded if they had Royal Marsden Stage I or IM disease, pure seminoma or primary mediastinal disease. Patients were also excluded if residual disease was deemed inoperable (e.g. bony metastases) or if serological or radiological progression occurred prior to planned post-treatment surgery.

All patients received platinum-based chemotherapy which was considered standard for that time or were entered in the Medical Research Council (UK) Testicular Cancer chemotherapy trials. Decisions regarding post-treatment surgery or surveillance were based upon CT scanning within 4 weeks of the final cycle of chemotherapy. Residual disease in the observation group was considered to be radiological abnormality in the region of previous measurable tumours which persisted for at least two post-treatment scans at least 8 weeks apart. If immediate surgery was proposed then this was usually performed within 8 weeks of the final cycle of chemotherapy.

### Study design

This study was a retrospective analysis of progression-free (PFS) and overall (OS) survival of patients meeting the inclusion criteria. The aim of the study was to determine the outcome of those patients in this group who underwent post-treatment surgery and of those who were subjected to a policy of 'expectant' surveillance. Patients who had a policy of surveillance tended to have had a TD-negative primary and had post-chemotherapy residual disease of ≤ 2 cm maximum diameter which were not cystic on CT scanning. There were variations from these guidelines according to patient and physician preference.

On meeting the inclusion criteria the patients were divided into two groups:

Group 1 – Surgery: patients who achieved a serological complete response (or whose markers remained normal) and who were left with potentially resectable radiological abnormalities who underwent immediate (within 8 weeks) surgery. Surgery usually consisted of RPLND, metastatectomy, or both.

Group 2 – Observation: patients who achieved a serological complete response (or whose markers remained normal) and who were left with potentially resectable radiological abnormalities who had a policy of active surveillance without surgery.

Study end-points for each patient were date of death or date of progression. For those who had not died or progressed the censure date was date of last contact (either clinic visit or tumour marker assessment).

### Statistical analysis

Kaplan–Meier (Kaplan and Meier, 1958) survival and progression-free survival curves were calculated for each group. Overall survival was defined from the date of histological diagnosis of cancer to date of death or last follow-up evaluation. Progression-free survival was defined from the date of histological diagnosis of cancer to date of serological or radiological progression (whichever was earlier) or last follow-up evaluation. Comparisons were evaluated for significance using the Cox–Mantel log-rank test (Peto et al, 1977).

### RESULTS

342 patients with testicular cancer were diagnosed in the area served by the regional Cancer Registary Office or who referred to Mount Vernon Hospital during the period January 1983 to January 1998. 183 had undergone platinum-based chemotherapy. From a review of this database 77 patients fitted the eligibility criteria. One of these patients was excluded as the hospital notes had been destroyed. The 76 remaining patients were divided into two groups based upon post-chemotherapy surgery or observation. The patient characteristics are given in Table 1.

### Group 1 – Surgery

Forty-eight patients achieved serological CR but had residual resectable disease and subsequently underwent surgery. Individual patient characteristics and the histological assessment of the residual disease is shown in Table 2. In the resection specimen

**Table 1** Patient demographics

| | Group 1 – Surgery | Group 2 – Observation |
|---|---|---|
| Patients (*n*) | 48 | 28 |
| Median age at diagnosis (years) | 28 | 27 |
| Range | 16–49 | 18–39 |
| Median follow-up (months) | 66 | 79 |
| Range | 13–153 | 20–172 |

© 2000 Cancer Research Campaign

**Table 2** Surgical group

| Patient number | Age at diagnosis | Stage at diagnosis | IGCCCG prognostic group | Histology of primary | Size of residual after chemotherapy | Histology of resection specimen |
|---|---|---|---|---|---|---|
| 1 | 34 | IIIc | Good | MTI | 5 cm | TD |
| 2 | 31 | IIa | Good | MTU | 1 cm | Necrosis |
| 3 | 26 | IVL3 | Good | MTU | 13 cm | Necrosis |
| 4 | 29 | 1° retroperitoneal | Good | MTU | 5 cm | TD |
| 5 | 28 | IIb | Intermediate | MTI | 2 cm | Necrosis |
| 6 | 33 | IIc | Poor | MTU | 9 cm | Carcinoma |
| 7 | 21 | IIc | Good | MTU | 2 cm | Necrosis |
| 8 | 21 | IIc | Good | MTU | 2.5 cm | TD |
| 9 | 24 | IIc | Good | MTI | 8 cm | MTI |
| 10 | 35 | IIa | Good | MTI | 3.5 cm | TD |
| 11 | 33 | IIIc | Intermediate | MTI | 18 cm | TD |
| 12 | 30 | IIIc | Poor | MTU | 7 cm | Necrosis |
| 13 | 38 | IIIc | Good | MTI | 4 cm | Necrosis |
| 14 | 27 | IIIc | Intermediate | MTI | 7 cm | Necrosis |
| 15 | 16 | IIc | Good | MTI | 3 cm | TD |
| 16 | 29 | IIa | Good | MTI | 2 cm | TD |
| 17 | 31 | IIa | Good | MTI | 2 cm | TD |
| 18 | 22 | IIIc | Good | MTI | 7 cm | MTI |
| 19 | 26 | IIa | Good | MTI | 1.5 cm | TD |
| 20 | 24 | I relapse IIa | Good | MTI | 11.2 cm | TD |
| 21 | 36 | IIb | Good | MTU | 1.3 cm | Necrosis |
| 22 | 21 | IIc | Good | MTI | 10 cm | TD |
| 23 | 23 | IVL3 | Poor | MTT | 4 cm | Necrosis + choriocarcinoma (as hCG immunostaining positive) |
| 24 | 28 | IIb | Good | MTI | 5 cm | TD |
| 25 | 35 | IIb | Good | MTI | 1.8 cm | TD |
| 26 | 31 | IIb | Good | MTI | 3 cm | TD |
| 27 | 19 | IV | Poor | MTI | 8 cm | TD |
| 28 | 23 | IVL3 | Poor | MTU | 4 cm | TD |
| 29 | 26 | IVL2 | Good | MTU | 13 cm | TD |
| 30 | 32 | IVL3 | Intermediate | MTT | 3 cm | Necrosis |
| 31 | 34 | IV | Poor | MTU | 6 cm | Necrosis |
| 32 | 49 | IVL1 | Good | MTI | 1.8 cm | MTI (+ immature adipose tissue) |
| 33 | 21 | IVL3 | Good | MTU | 2 cm | TD + sarcoma |
| 34 | 28 | IVL3 | Poor | MTU | 3 cm | TD |
| 35 | 20 | IVL3 | Intermediate | MTU | 1.5 cm | Necrosis |
| 36 | 18 | IVL3 | Intermediate | MTI | 8 cm | TD |
| 37 | 44 | IIc | Good | MTI | 5 cm | TD |
| 38 | 19 | IVL3 | Poor | MTI | 1.5 cm | TD |
| 39 | 22 | IV H+ | Poor | MTU | 10 cm | Necrosis |
| 40 | 29 | IVL3 | Good | MTI | 3 cm | Necrosis |
| 41 | 37 | IVL3 | Poor | MTI | 5 cm | Necrosis + yolk sac tumour |
| 42 | 38 | IIc | Good | MTU | 1.5 cm | TD + myxoid yolk sac tumour |
| 43 | 27 | IIc | Intermediate | MTI | 2.5 cm | TD |
| 44 | 23 | IVL2 | Good | MTU | 1.3 cm | Necrosis |
| 45 | 21 | IIb | Good | MTU | 0.8 cm | Necrosis |
| 46 | 39 | IIc | Intermediate | MTU | 13 cm | TD |
| 47 | 37 | IVL3 | Intermediate | MTU | 10 cm | TD |
| 48 | 37 | I relapse IIa | Good | MTU | 2 cm | TD |

IGCCCG = International Germ Cell Cancer Collaborative Group; MTI = malignant teratoma, intermediate; MTT = malignant teratoma, trophoblastic; MTU = malignant teratoma, undifferentiated; hCG = human chorionic gonadatrophin; TD = differentiated teratoma. Size was determined as the maximum tumour diameter in any plane. Histology is according to the classification of Pugh (1976)

50% of patients had TD or a combination of TD and necrotic tissue/fibrosis, 31.25% had necrotic tissue/fibrosis only and 18.75% had residual active tumour.

Four of these 48 patients have subsequently relapsed and died from progressive NSGCT. Of these, three had active tumour in the resection specimen and the other had necrosis only. Four others relapsed radiologically but were found to have TD at subsequent resection. Two others relapsed with active NSGCT but have been rendered disease-free with salvage chemotherapy and surgery (RPLND). One further patient relapsed with a second primary testicular cancer (seminoma).

### Group 2 – Observation

Twenty-eight patients achieved a serological complete response but had residual radiological abnormalities which were deemed potentially operable. The patient characteristics of this group are shown in Table 3. Three patients (numbers 5, 19 and 28) had residual radiological masses < 1 cm in diameter (considered within the normal range for lymph nodes on CT) but were included in this analysis as these abnormalities were thought to be in the same area as previous tumour deposits and have persisted for the entire follow-up period.

*© 2000 Cancer Research Campaign*

**Table 3** Observation group. Three patients had residual disease > 3 cm. Patient 26 refused surgery. The residual disease in patients 6 and 27 showed incomplete resolution on subsequent CT scanning

| Patient number | Age at diagnosis | Stage at diagnosis | IGCCCG prognostic group | Histology of primary | Size of residual after chemotherapy | Histology of resection specimen if relapsed |
|---|---|---|---|---|---|---|
| 1 | 31 | IIb | Good | MTI | 1 cm | |
| 2 | 39 | IVL2 | Good | MTU | 2 cm | |
| 3 | 35 | IVL3 | Intermediate | MTU | 1 cm | |
| 4 | 27 | IIb | Intermediate | MTI | 1 cm | |
| 5 | 32 | IIa | Good | MTU | 0.3 cm | |
| 6 | 23 | IIb | Good | MTU | 2.5 cm | |
| 7 | 19 | IVL3 | Poor | MTI | 1.5 cm | |
| 8 | 38 | IIc | Good | MTU | 1.5 cm | |
| 9 | 27 | IIc | Good | MTU | 1.5 cm | TD |
| 10 | 23 | IIc | Poor | MTU | 1.8 cm | |
| 11 | 34 | IV | Poor | MTU | 2 cm | |
| 12 | 32 | IVL3 | Poor | MTT | 1 cm | |
| 13 | 18 | IVL3 | Intermediate | MTI | 2 cm | Choriocarcinoma |
| 14 | 27 | IIb | Good | MTU | 1.5 cm | |
| 15 | 34 | IIc | Intermediate | MTI | 2 cm | TD |
| 16 | 23 | IIc | Good | MTI | 2 cm | |
| 17 | 21 | IVL3 | Intermediate | MTU | 1 cm | |
| 18 | 27 | IVL3 | Good | MTU | 1 cm | |
| 19 | 33 | IVL3 | Intermediate | MTU | 0.8 cm | |
| 20 | 35 | IVH+ | Poor | MTU | 1 cm | |
| 21 | 36 | III | Good | MTT | 1 cm | MTI |
| 22 | 26 | IIb | Good | MTU | 2 cm | MTU |
| 23 | 24 | IVL1 | Good | MTI | 2 cm | |
| 24 | 27 | IIIb | Good | MTU | 2 cm | |
| 25 | 37 | IVL3 | Intermediate | MTU | 2 cm | |
| 26 | 21 | IVL1 | Intermediate | MTI | 3 cm | |
| 27 | 31 | IVL3 | Good | MTU | 3 cm | |
| 28 | 27 | IIb | Good | MTU | 0.6 cm | |

IGCCCG = International Germ Cell Cancer Collaborative Group; MTI = malignant teratoma, intermediate; MTT = malignant teratoma, trophoblastic; MTU = malignant teratoma, undifferentiated; TD = differentiated teratoma. Histology is according to the classification of Pugh (1976)

Of these 28 patients, two have subsequently died, one from relapsed NSGCT and one from a motor-vehicle accident. A total of six patients have relapsed, of whom five had relapse chemotherapy and four of these went on to have surgery. One patient (number 13) had surgery alone. The histology of the resected tissue in those patients who went to surgery is shown in Table 3. Relapses have occurred in two of eight patients with TD in the primary tumour and in four of 20 patients without TD in the primary tumour.

Two of these patients who relapsed but are now disease-free are interesting. Both had TD in the primary testicular cancer. One had residual disease around the (L) brachial plexus which at initial thoracotomy was deemed unresectable, but after later marker relapse and salvage chemotherapy, successful resection of residual active disease was performed at a surgical unit specializing in brachial plexus injuries. The other patient had initial RMH Stage IV malignant teratoma trophoblastic (MTT) and had resection of residual necrotic tissue and TD from para-aortic lymph nodes. A small peripheral lung lesion was seen at the initial post-treatment staging CT scan but the patient was observed because of his fragile psychological state at that time. This patient subsequently had a marker relapse some 18 months later. CT and positron-emission tomographic (PET) scanning suggested that the lung lesion was the sole site of disease. Resection of this nodule showed active choriocarcinoma. He has been observed without further chemotherapy and is now disease-free some 20 months later.

Six further patients who had TD in the primary testicular cancer had residual radiological changes and avoided surgery. Two of these had radiological changes which resolved over periods of 6 and 12 months (patients number 16 and 17). Four patients continue to have minor radiographic changes (patients number 1, 4, 7 and 26). One patient (number 26) refused surgery.

Comparisons between the two groups in terms of initial stage, prognostic group and histology is given in Table 4. Patients with TD in the primary tumour were more likely to have had surgery rather than observation.

**Table 4** Patient characteristics according to initial stage, histology and size of residual tumour

| | Surgery | Observation |
|---|---|---|
| % RMH stage | | |
| Stage II | 52 | 43 |
| Stage III | 10.5 | 7 |
| Stage IV | 37.5 | 50 |
| % IGCCCG prognostic group | | |
| Good | 60.5 | 53.5 |
| Intermediate | 18.5 | 28.5 |
| Poor | 21 | 18 |
| % Histology of primary | | |
| MTT | 4 | 7 |
| MTU | 44 | 64 |
| MTI | 52 | 29 |
| Average size residual mass (largest) | 5.4 cm | 1.6 cm |

*© 2000 Cancer Research Campaign*

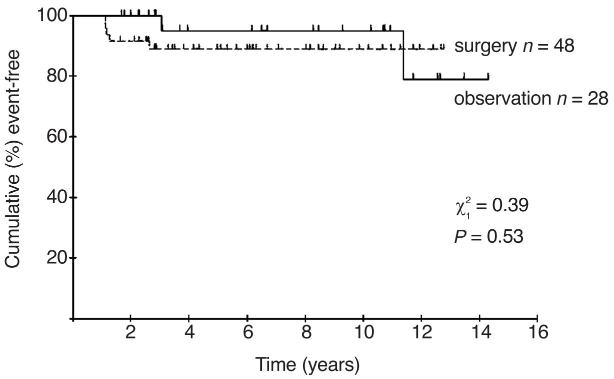

**Figure 1** Overall survival

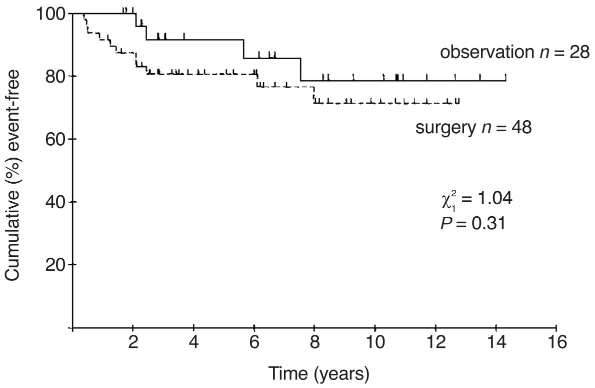

**Figure 2** Progression-free survival

### Overall survival and progression-free survival

Kaplan–Meier estimates of overall survival were calculated for each group and the survival curves are shown in Figure 1. There was no significant difference in survival between the two groups ($P$ = 0.53; Cox–Mantel log-rank). Similar progression-free survival curves are shown in Figure 2. Again there was no significant difference between the two groups ($P$ = 0.31; Cox–Mantel log-rank).

An attempt to correlate individual cause-specific deaths was made with initial International Germ Cell Cancer Collaborative Group (IGCCCG, 1997) prognostic group (patients were allocated a prognostic group retrospectively). The results are shown in Table 5. Because of the small number of deaths in each group there was no significance in the result.

### Overall survival in surgical group with respect to resection histology

Figure 3 shows the overall survival in the surgical group according to histology of the resected specimen. As expected, the finding of active NSGCT was associated with a significantly worse prognosis ($P$ = 0.02; Cox–Mantel log-rank) than in those patients who had differentiated teratoma or necrosis/fibrosis only.

### DISCUSSION

Since the advent of platinum-based chemotherapy, the outlook for patients with NSGCT has been extremely favourable. Post-chemotherapy surgery is often helpful in achieving cure. It is also useful in eradicating residual TD and in guiding clinicians as to whether further chemotherapy is appropriate. The most common surgical approach, RPLND, is not without risk, however, and long-term morbidity is common even in experienced hands. The histology of resected residual tissue in the surgery group presented here (differentiated teratoma 50%, necrosis/fibrosis 31.75%, active cancer 18.75%) is similar in content to two recently published large combined series (Steyerberg et al, 1997; Stenning et al, 1998). Overall and progression-free survival curves were also similar.

Whether this surgical approach should be offered to all patients regardless is currently a matter of some debate. The utility of the germ cell tumour markers αFP and βhCG must also be taken into consideration. Most clinicians would not now routinely offer RPLND in patients with both radiological and serological

**Table 5** Deaths according to initial prognostic group

| | Deaths in surgical group | Deaths in observation group |
|---|---|---|
| IGCCCG prognostic group | | |
| Good | 2/29 | 0/15 |
| Intermediate | 1/9 | 0/8 |
| Poor | 2/10 | 1/5 |

complete responses. There are, however, a group of patients who have serologic complete responses but with residual radiographic abnormalities and these patients always give cause for concern. Of these, the patients with TD in the primary tumour are an important sub-group, as incomplete resection of TD may lead to late relapse in the form of de-differentiated tumour (Christmas et al, 1998*b*).

Despite the importance of this group of patients there have been relatively few publications dealing with this issue. The Indiana group (Debono et al, 1997) have recently reported their experience with disseminated NSGCT. Among the groups of patients reported they described 10 patients with TD-positive primary tumours with residual radiologic abnormalities in the face of normal serum tumour markers. In nine of these patients the radiological abnormalities subsequently resolved and in only one has there been relapse (in a separate site). A separate group of 27 patients with TD-negative primary tumours with residual radiological abnormalities was also described. Of these patients three progressed at the site of residual radiological abnormality within 10 months and one relapsed after 4 years with disseminated disease. The radiological abnormalities in the other 23 all resolved but two have relapsed subsequently (both at the sites of previous disease).

Jones et al (1998), described 101 patients with normal serum tumour markers at the end of chemotherapy with at least one residual mass under close observation and no malignant teratoma resected from other sites. In this group 35 have subsequently progressed, and of these 23 have died from progressive NSGCT and three have died from other causes. In the group without subsequent progression five have died from non-cancer causes. There was little difference in the chance of progression whether the remaining radiological abnormality was in the lung or abdomen. There was also no clear predictive value of either pre- or post-chemotherapy mass size on the chances of relapse. The risk of relapse was, however, higher in patients with more than one site of

© 2000 Cancer Research Campaign

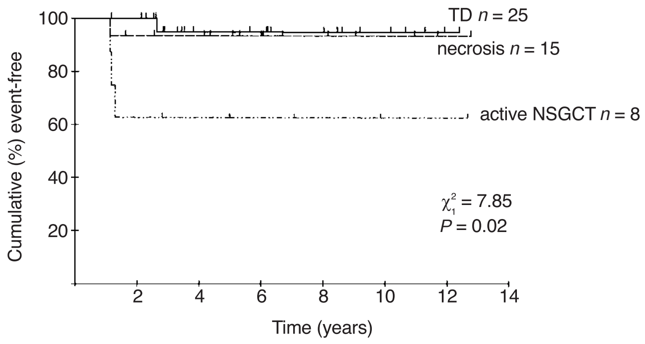

**Figure 3** Overall survival according to histology of resected mass. TD = differentiated teratoma; NSGCT = non-seminomatous germ cell tumour residual radiological abnormality. Radiotherapy was not routinely given to residual masses in this study except at one centre. At this centre there was no involved site progression noted in any of 19 patients treated, however in none of these reported patients was there complete regression seen with radiotherapy.

In our series of 28 patients with residual radiological abnormalities, six have subsequently relapsed and one patient has died of NSGCT. The patient who died relapsed in multiple sites. The other relapses were in sites of known residual masses (four in retroperitoneal lymph nodes and one in lung). Four of these patients have been effectively salvaged with a combination of surgery (RPLND) and chemotherapy (two had TD and two had active NSGCT in the resected specimen), and one other has been effectively salvaged with surgery alone (choriocarcinoma in the resected specimen). The overall survival in this group is excellent. 22 of 28 patients (79%) thus avoided potentially morbid surgery.

Of some interest in this group is the low rate of radiographic resolution of residual tumour mass in patients who had TD in the primary cancer. Of these eight patients only two had radiographic resolution, albeit incomplete (both had residual disease > 2 cm; patients number 6 and 27 in Table 3), and two had disease progression. This is compared with the Indiana experience in which nine of 10 residual masses resolved (Debono et al, 1997). Our results are more in line with the results from the UK Medical Research Council's TE16 trial (Jones et al, 1998), where the spontaneous regression rates were 33% in lung and 39% in retroperitoneal nodes.

Our results suggest that residual radiographic abnormalities (size ≤ 2 cm) in patients with complete serologic responses can be safely observed without detriment in terms of progression-free and overall survival. Of course, the two groups are not evenly matched in terms of residual mass size (only three patients in the observation group had residual radiologic abnormalities > 2 cm and one of these refused surgery) or the presence or absence of TD in the primary (interestingly they were well matched in terms of initial Royal Marsden stage and IGCCCG prognostic group, Table 3). Correctly selecting patients who would benefit from surgery would obviously remove from the observation group those at greatest risk of relapse. Steyerberg et al (1998) recently examined the principles of observation and resection in patients with residual masses < 2 cm. They concluded that with currently available 'evidence' (i.e. data from resection histology of small masses: 4% had cancer if the residual mass was 0–1 cm, and 7% had cancer if the residual mass was 1–2 cm in size) a randomized clinical trial to 'answer' this important question would never be feasible. For a survival advantage of just 3% at 5 years, at a conservative estimate 2800 patients would need to be randomized. For an observational study it is possible that even greater numbers of patients will be needed to overcome an uneven distribution in patients. The same authors, using a decision analysis model, tested this assumption and estimated a 5-year survival advantage of 2.7–4.3% for resection of masses < 2 cm in all cases (Steyerberg et al, 1999).

Laparoscopic retroperitoneal lymphadenectomy might prove to be a useful and less morbid alternative to open surgery in selected patients. Janetschek et al (1999) have recently published their results in 24 patients submitted to this procedure. The perioperative complication rate was low and anterograde ejaculation was preserved in all cases. Of interest is that of the eight residual masses < 1 cm which were resected, seven contained necrotic tissue only.

At present it remains reasonable that all patients with residual masses at the completion of chemotherapy should be considered for surgery, but there is a smaller subset in whom close observation could be considered. If these patients are deemed suitable candidates for observation then they could be spared the morbidity of surgery. The usefulness of new scanning modalities such as PET to distinguish between active tumour and necrosis has yet to be confirmed. Laparoscopic retroperitoneal lymphadenectomy might also prove to be a useful less-morbid procedure in selected patients. Whether these techniques can be used to safely defer radical surgery is a question for the future.

### ACKNOWLEDGEMENTS

We are grateful to J Bradley, H Parkhouse and E Townsend for the resection of residual masses in some of these patients.

### REFERENCES

Aass N, Klepp O, Cavallin-Shahl E, Dahl O, Wicklund H, Unsgaard B, Ahlstrom S and Fossa SD (1991) Prognostic factors in unselected patients with nonseminomatous testicular cancer: a multivariate analysis. *J Clin Oncol* **9**: 818–826

Bajorin DF, Herr H, Motzer RJ and Bosl GJ (1992) Current perspectives of the role of adjunctive surgery in the combined modality therapy for patients with germ cell tumours. *Semin Oncol* **19**: 148–158

Christmas TJ, Doherty AP, Rustin GJS, Seckl MJ and Newlands ES (1998*a*) Excision of residual masses of metastatic germ cell tumours after chemotherapy: the role of extraperitoneal surgical approaches. *Br J Urol* **81**: 301–308

Christmas TJ, Smith GL and Kooner R (1998*b*) Vascular interventions during post-chemotherapy retroperitoneal lymph-node dissection for metastatic testis cancer. *Eur J Surg Oncol* **24**: 292–297

Debono DJ, Heilman DK, Einhorn LH and Donohue JP (1997) Decision analysis for avoiding post chemotherapy surgery in patients with disseminated nonseminomatous germ cell tumours. *J Clin Oncol* **15**: 1455–1464

Donohue JP and Rowland RG (1984) The role of surgery in advanced testicular cancer. *Cancer* **54**: 2716–2721

Donohue JP, Rowland RG, Kopecky K, Steidle CP, Geiger G, Ney KG, Williams S and Loehrer P (1987) Correlation of computerised tomographic changes and histological findings in 80 patients having radical retroperitoneal lymph node dissection after chemotherapy for testis cancer. *J Urol* **137**: 1176–1179

Fossa SD, Aass N, Ous S, Hoie J, Stenwig AE, Lien HH, Paus E and Kas P (1989) Histology of tumour residuals following chemotherapy in patients with advanced non-seminomatous testicular cancer. *J Urol* **142**: 1239–1242

Fossa SD, Qvist H, Stenwig AE, Lien HH, Ous S and Giercksky KE (1992) Is post chemotherapy retroperitoneal surgery necessary in patients with non-seminomatous germ cell tumour and minimal residual tumour masses? *J Clin Oncol* **10**: 569–573

Gelderman WA, Koops HS, Sleijfer DT, Oosterhuis JW and Oldhoff J (1986) Treatment of retroperitoneal residual tumour after PVB chemotherapy of non-seminomatous testicular tumours. *Cancer* **58**: 1418–1421

*© 2000 Cancer Research Campaign*

Hendry WF, A'Hern RP, Hetherington JW, Peckham MJ and Dearnaley DP (1993) Paraaortic lymphadenectomy after chemotherapy for metastatic non-seminomatous germ cell tumours: prognostic value and therapeutic benefit. *Br J Urol* **71**: 208–213

Horwich A, Mason MD and Hendry F (1995) Testicular tumours. In *Oxford Textbook of Oncology*, Peckham M, Pinedo H, Veronesi U (eds) pp 1407–1438. Oxford University Press: Oxford

International Germ Cell Cancer Collaborative Group (1997) International Germ Cell Consensus Classification: a prognostic factor-based staging system for metastatic germ cell cancers. *J Clin Oncol* **15**: 594–603

Jaeger N, Weissbach L and Bussar-Maatz R (1994) Size and status of metastases after inductive chemotherapy of germ cell tumours. Indications for salvage operation. *World J Urol* **12**: 196–199

Janetschek G, Hobisch A, Hittmair A, Holtl L, Peschel R and Bartsch G (1999) Laparoscopic retroperitoneal lymphadenectomy after chemotherapy for stage IIB nonseminomatous testicular carcinoma. *J Urol* **161**: 477–481

Jones WG, Stenning SP and Read G (1998) Long term follow up of unresected residual masses following platinum based chemotherapy for metastatic non-seminoma of the testis (MRC Study TE16): a preliminary report. In *Germ Cell Tumours IV*, Jones WG, Appleyard I, Harnden P, Joffe JK (eds) pp 269–275. John Libbey and Co Ltd: London

Kaplan E and Meier P (1958) Nonparametric estimation from incomplete observations. *J Am Stat Assoc* **53**: 457–481

Levitt MD, Reynolds PM, Sheiner HJ and Byrne MJ (1985) Non-seminomatous germ cell tumour: residual masses after chemotherapy. *Br J Surg* **72**: 19–22

Peto R, Pike MC, Armitage P, Breslow NE, Cox DR, Howard SV, McPherson K, Peto J and Smith PG (1977) Design and analysis of randomised clinical trials requiring prolonged observation of each patient. *Br J Cancer* **35**: 1–39

Pugh RCB (1976) Testicular tumours. In *Pathology of the Testis*, Pugh RCB (ed) pp 149–159. Blackwell Science: Oxford

Rabbani F, Gleave ME, Coppin CM, Murray N and Sullivan LD (1996) Teratoma in primary testis tumour reduces complete response rates in retroperitoneum after primary chemotherapy. The case for primary retroperitoneal lymph node dissection of Stage IIb germ cell tumours with teratomatous elements. *Cancer* **78**: 480–486

Sagalowsky AI, Ewart DH, Molberg K and Peters PC (1990) Prediction of mass histology after chemotherapy for advanced testis cancer. *Urology* **36**: 537–542

Stenning SP, Parkinson MC, Fisher C, Mead GM, Cook PA, Fossa SD, Jones WG, Newlands ES, Oliver RT, Stenwig AE and Wilkinson PM (1998) Residual masses post-chemotherapy for germ cell tumour: content, clinical features and prognosis. *Cancer* **83**: 1409–1420

Steyerberg EW, Keizer HJ, Zwartendijk J, Van Rijk GL, van Groeninge S, Habbema JD and Stoter G (1993) Prognosis after resection of residual masses following chemotherapy for metastatic non-seminomatous testicular cancer: a multivariate analysis. *Br J Cancer* **68**: 195–200

Steyerberg EW, Keizer HJ, Stoter G and Habbema JDF (1994) Predictors of residual mass histology following chemotherapy for metastatic non-seminomatous testicular cancer: a quantitative overview of 996 resections. *Eur J Cancer* **30**: 1231–1239

Steyerberg EW, Keizer HJ, Fossa SD, Sleijfer DT, Toner GC, Schraffer H, Mulders PF, Messemer JE, Ney K and Donohue JP (1995) Prediction of residual mass histology after chemotherapy for metastatic non-seminomatous germ cell tumour: multivariate analysis of individual patient data from six study groups. *J Clin Oncol* **13**: 1177–1187

Steyerberg EW, Keizer HJ, Messener JE, Toner GC, Schraffordt Koop SD, Gerl A, Sleijfer DT, Donohue JP and Habbema JD (1997) Residual pulmonary masses after chemotherapy for non-seminomatous germ cell tumour: prediction of histology. *Cancer* **79**: 345–355

Steyerberg E, Keizer HJ and Habbema JDF (1998) Resection of small residual masses and evidence-based medicine. In *Germ Cell Tumours IV*, Jones WG, Appleyard I, Harnden P, Joffe JK (eds) pp 261–268. John Libbey and Co Ltd: London

Steyerberg EW, Marshall PB, Keizer HJ and Habbema JD (1999) Resection of small, residual retroperitoneal masses after chemotherapy for nonseminomatous testicular cancer: a decision analysis. *Cancer* **85**: 1331–1341

Stomper P, Kalish L, Garnick M, Richie JP and Kantoff PW (1991) CT and pathologic predictive features of residual mass histologic findings after chemotherapy for non-seminomatous germ cell tumours: can residual malignancy or teratoma be excluded? *Radiology* **180**: 711–714

*© 2000 Cancer Research Campaign*
